# Monitoring HIV Epidemic in Pregnant Women: Are the Current Measures Enough?

**DOI:** 10.1155/2015/194831

**Published:** 2015-01-19

**Authors:** Purva Sarkate, Supriya Paranjpe, Nayana Ingole, Preeti Mehta

**Affiliations:** Department of Microbiology, Seth G. S. Medical College and KEM Hospital, Mumbai 400012, India

## Abstract

*Introduction.* Burden of HIV in pregnant women follows overall epidemic in India. Hence, it is imperative that prevalence calculations in this group be accurate. The present study was carried out to determine prevalence of HIV in pregnant women attending our hospital, to determine trend of HIV infection and to compare our results with reported prevalence. *Methods.* All pregnant women are routinely counselled for HIV testing using opt-out strategy. Year-wise positivity and trend were determined in these patients over a period of five years. The positivity in different age groups was determined. *Results.* 31,609 women were tested of which 279 (0.88%) were positive. Positivity showed a declining trend over study period and significant quadratic trend (biphasic, *P* < 0.05) was observed. The positivity in older age group ≥35 years (1.64%) was significantly more than younger age groups (0.76% in 15–24-year and 0.94% in 25–34-year age group) (*P* = 0.0052). *Conclusion.* A significant decline in HIV positivity was seen over the study period. Taking into account heterogeneous nature of HIV epidemic even within the same district, analysis at local levels especially using the prevention of parent to child transmission of HIV program data is critical for HIV programming and resource allocation.

## 1. Introduction

In India, the first case of human immunodeficiency virus (HIV) was reported in 1986 in Chennai [1]. The country experienced a sharp increase in the estimated number of HIV infections, from a few thousand in the early 1990s to around 5.2 million adults and children living with HIV/AIDS in 2005 [2]. In 1987, a National AIDS Control Programme (NACP) was launched under the Ministry of Health and Family Welfare, Government of India, to coordinate national responses. Its activities covered surveillance, blood screening, and health education [3].

Mother to child transmission (MTCT) is the most important source of HIV in children less than 15 years of age. To prevent mother to child transmission of HIV, prevention of parent to child transmission of HIV (PPTCT) programme had been launched under the NACP. This programme is the largest national antenatal screening programme in the world [4].

Burden of HIV in pregnant women and children follows overall HIV epidemic in India. India has a concentrated HIV epidemic and accordingly the diagnostic and treatment services are concentrated in states and districts having high HIV prevalence. These services are limited in the rest of the country [5]. Hence, it is imperative that the prevalence calculations be accurate. Various studies have been carried out to evaluate the utility of different surveillance methodologies used to estimate the prevalence of HIV [6–10].

Ours is a tertiary care referral centre located in Mumbai, western India, and has been a part of PPTCT since its inception. Ours was also a centre of excellence for the AZT (zidovudine) feasibility study before PPTCT was launched. We cater to almost 600 to 800 pregnant women per month.

The present study was carried out to determine the prevalence of HIV in pregnant women attending our hospital, to determine the trend of HIV infection in them over a period of five years, and to see if our results are comparable to reported prevalence in India.

## 2. Methods

Retrospective analysis of data was carried out over a period of five years from January 2008 to December 2012 in the department of microbiology at a tertiary care referral centre in Mumbai, western India, after obtaining institutional review board permission. Under the PPTCT program, all pregnant women are routinely counseled for HIV testing. Opt-out strategy is followed. 3–5 mL of venous blood sample was collected in a sterile plain container from all pregnant women after written informed consent. The samples were tested as per Strategy III of National AIDS Control Organization (NACO) guidelines [11]. As per this strategy, three serial antibody tests are performed to label a patient/client as positive for HIV. Specimens which were reactive by the screening test and nonreactive by one of the supplemental tests were reported as indeterminate and sent to national reference laboratory for confirmation by western blot (WB). The results of western blot test were considered as final in these indeterminate cases.

Analysis was done to calculate the year-wise positivity and trend was determined. Women were divided into age groups of 15–24, 25–34, and 35–49 years. The positivity in the different age groups was compared using chi-square test. Trend analysis was done using Joinpoint Regression Program (4.1.1.1).

## 3. Results

**Figure 1:**
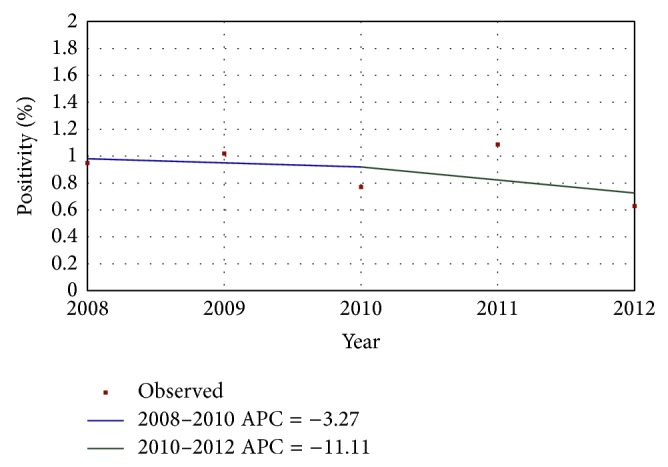
HIV positivity trend over a period of five years. The annual percent change (APC) is significantly different from zero at alpha = 0.05.

A total of 31,609 women were tested over a period of five years. 279 were positive for HIV antibodies giving an overall positivity rate of 0.88% (95% CI, 0.778–0.99). The year-wise samples tested, HIV positivity, and number of indeterminate samples have been depicted in Table 1. The positivity showed a declining trend over the study period and a significant quadratic trend (biphasic, *P* < 0.05) was observed. The average annual percentage decline was 3.27 from 2008 to 2010 and 11.11 from 2010 to 2012 (Figure 1).

Of the 31,600 pregnant women (excluding those with indeterminate result for further analysis), 45.72%, 50.61%, and 3.67% were in the age group of 15–24, 25–34, and 35–49 years, respectively.

The positivity in older age group (1.64%) was significantly more than younger age groups (0.76% in 15–24-year and 0.94% in 25–34-year age group) (*P* = 0.0052) (Table 2).

Only nine of the 31,609 (0.029% CI, 0.01–0.06) samples had indeterminate result and eight could be sent for western blot. Two of these samples (25%) were negative by western blot and six (75%) were indeterminate.

## 4. Discussion

HIV infection in women occurs primarily during their reproductive years. Thus pregnancy provides a unique opportunity for implementing prevention strategies against HIV infection. Opt-out approach is followed for HIV testing in antenatal care clinic (ANC) women and the refusal for testing is less than 1%. Hence, the degree of bias in estimating the prevalence is negligible.

In the present study positivity in the older age group (1.64%) was significantly higher. Gupta et al. from New Delhi reported a higher positivity in the younger age group [12]. It may be because their study was carried out from January 2003 to December 2006 and since then there has been an overall reduction in HIV prevalence in India [13]. Majority of women in the general population acquire HIV infection from their infected partners through heterosexual route and longer exposure leads to higher risk [14, 15]. There is also increased awareness about the disease and methods of its prevention in the general population in the recent years. All these factors may have contributed to lesser positivity in the younger age group. However, with our study being retrospective, information about other demographic profiles of the study population was not available and is a limitation of the study.

In India, under NACP III, western blot is the only option available to resolve cases which are indeterminate by rapid tests. Western blot could be done for 8 of the 9 indeterminate samples. The results of WB were indeterminate in 6 (75%) and negative in 2 (25%) samples. The samples had to be sent to the higher reference laboratory for WB, the result of which took up to two weeks. The prime concern with WB indeterminate result as seen in 75% of patients in the present study is obviously the derived uncertainty in decision making for further management of these patients. Currently, these patients have no other choice left than to do repeat testing after three months. However, the clients when asked to come for repeat testing, even after pre- and posttest counseling, are often lost to follow-up. Also, they often register in the second or third trimester and deliver before three months. Moreover, as per Indian culture, many pregnant females go to their maternal place for delivery which makes it further difficult to trace them. Under the PPTCT program there are no set guidelines for procedure in case of consistent indeterminate results in pregnancy or in women with indeterminate result presenting directly in labour. Hence, tests which can resolve this indeterminate status will go a long way not only in protecting the unborn baby against HIV infection by timely prophylaxis but also in alleviating the stress of the woman and her family during her pregnancy. Under NACP, DNA PCR is available for free for early infant diagnosis of infants born to HIV positive mothers. Efforts can be made at the national level to make this test available for ANC mothers with indeterminate results free of cost.

In India, the HIV prevalence among ANC attendees has reduced gradually from about 0.9% in 2003-2004 to 0.35% in 2012-2013 [13]. However, the HIV positivity at our centre is higher than the national prevalence (Table 1). Mumbai is the capital of the Indian state of Maharashtra. The city is one of the most populous cities in the world with a population of 1,19,78,450. Along with the neighbouring suburbs of Navi Mumbai and Thane, it forms the world's 4th largest urban agglomeration. The number of migrants into Mumbai from outside Maharashtra during the 1991–2001 decade was 11.2 lakhs, which amounted to 54.8% of the net addition to the population of Mumbai [16].

Ours is a tertiary care centre in Mumbai which mainly caters to middle and low socioeconomic group. A lot of migrant populations dwell in the city of Mumbai. Being cut off from their families or social support networks and having limited access to prevention services provide opportunities to indulge in risky behaviour which might account for the higher positivity [17].

Mumbai has been classified as a high prevalence district wherein the prevalence of HIV in general population is estimated to be consistently more than 1% as per ANC HIV sentinel surveillance (ANC-HSS) data from 2003 onwards [13]. In the present study, the positivity of 0.88% (95% CI, 0.78–0.99) is comparatively lower. Ours is a blood testing centre for ANC-HSS and not a sentinel site. Hence, the patients coming to our centre are not included in the ANC-HSS. This demonstrates that in the same city there may be different pockets with varying prevalence of the disease further highlighting the heterogeneity of the epidemic. This is especially important to note as the resources for HIV prevention, care, and support interventions are allocated, largely based on classification of districts as per prevalence.

Significant decline in HIV positivity has been reported by various other authors from across the country [18, 19] and ANC-HSS data [13]. Giri et al. in their study from Loni, Maharashtra, have seen a significant decline of 0.75% (2008) to 0.22% (2011) in ANC attendees. The present study also demonstrates a decline in HIV positivity; however, the decline is not as steep as reported by other studies. This may be because in Mumbai though the HIV epidemic has stabilized it may not be declining because of the high influx of migrant population every year.

India is a large country with 29 states, seven union territories, and about 674 districts. It is important to base resource allocations for HIV/AIDS programming on sound evidence. Availability of accurate measures of HIV prevalence, incidence, and their trends at national, regional, and local levels of programming will allow appropriate allocation of resources [10]. In view of our large population pool of one billion plus, a mere 0.1 percent increase in the prevalence rate will raise the number of persons living with HIV by over half a million [12].

Several methods have been proposed for measuring HIV prevalence; yet each presents specific challenges. Surveillance of HIV infection among pregnant women attending antenatal care clinics (ANC) has been the mainstay system of monitoring of HIV epidemic in India. Also, nationally representative population-based surveys (NPS) with HIV testing have also been used to estimate prevalence. However, each has its own limitations.

A major limitation of sentinel surveillance system is that it is conducted for a specific period once a year and has limited geographical coverage. Hence, sampling is often not representative of smaller and more remote areas in a country [7]. Secondly, small sample sizes (only 400 women are tested in a given district during a defined sampling period) result in wide confidence intervals and large variations in prevalence between years. As a result, trend analyses at the district level and subdistrict level using this dataset are not conclusive [10]. In fact, as in other countries [7], studies in India have also shown that ANC-HSS sites overestimate HIV prevalence in the general population [20, 21].

The main limitation of population-based surveys is potential bias arising from refusal of study subjects to participate in the survey or to have blood taken for HIV testing, as well as their absence at the time of survey [7].

India initially launched its PPTCT programme in 2002 in the high risk states and has now expanded it to include 410 districts of India [5]. Geographic coverage across the country is also being saturated by making PPTCT services increasingly available at primary health centres, community health centres, and selected private facilities through a public-private partnership model [10]. In December 2013, there were 15539 HIV counselling and testing centres in India [5]. In almost all government healthcare facilities including hospitals, ANC mothers are routinely offered HIV counselling and testing services using the opt-out strategy during their first antenatal visit. This routinely collected information is a great source to estimate prevalence of HIV in low risk group.

Advantages of PPTCT data include large sample sizes, lower levels of selection biases at the facility and participant level (as HIV test acceptance levels are high), routine data collection, and low additional cost for data collection [10]. Also, due to the large numbers tested, the confidence intervals around the estimates of HIV prevalence are narrower in the PPTCT data compared to the ANC-HSS data. Another major strength of PPTCT data is that, similar to population-based survey, HIV serostatus can be linked to social, behavioral, and other biomedical information, providing the opportunity to study the dynamics of the epidemic in more detail.

Several countries have explored the utility of MTCT data for HIV surveillance, and some such as Uganda and Thailand have replaced their unlinked anonymous testing data with MTCT data for surveillance due to higher coverage and participation [22, 23]. In the Indian context also, Kumar et al. and Sgaier et al. have recommended the use of PPTCT data in place of the annual HIV sentinel surveillance data for determining HIV prevalence and trends [10, 24]. They also concluded that analyses of HIV prevalence at the subdistrict level and trends at the district and subdistrict level are only possible with PPTCT and not HSS-ANC data.

To conclude, our hospital based study indicates a decreasing trend of HIV positivity in pregnant mothers. Taking into account this heterogeneous nature of HIV epidemic even within the same district, analysis at local levels is critical for HIV programming and resource allocation. Hence, PPTCT data with its various advantages and expanded coverage needs to be considered as an important source to determine the prevalence and trend in general population.

## Figures and Tables

**Table 1:** HIV positivity over a period of five years.

Year	Number Of samples tested	Number of positives	% positivity (95% CI)	Number of negatives	Number of indeterminate samples
2008	7290	69	0.95 (0.75–1.21)	7220	1
2009	4980	51	1.02 (0.77–1. 35)	4929	0
2010	6127	47	0.77 (0.57–1.03)	6079	1
2011	6233	68	1.09 (0.85–1.39)	6160	5
2012	6979	44	0.63 (0.46–0.85)	6933	2
Total	**31609**	**279 **	**0.88** **(0.78**–**0.99)**	**31321**	**9 (0.029%)**

**Table 2:** Age- and year-wise HIV positivity.

	15–24 yrs % (95% CI^*^)	25–34 yrs % (95% CI^*^)	35–49 yrs % (95% CI^*^)
2008 (*n* = 7290)			
HIV positives number	28	37	4
% positivity (95% CI)	0.86 (0.58–1.26)	0.98 (0.7–1.36)	1.70 (0.55–4.59)
Total	3269	3785	235
2009 (*n* = 4980)			
HIV positive	21	26	4
% positivity (95% CI)	0.91 (0.58–1.41)	1.04 (0.69–1.54)	2.22 (0.71–5.95)
Total	2309	2491	180
2010 (*n* = 6127)			
HIV positive	22	21	4
% positivity (95% CI)	0.77 (0.49–1.18)	0.69 (0.44–1.07)	1.71 (0.55–4.61)
Total	2849	3043	234
2011 (*n* = 6233)			
HIV positive	23	41	4
% positivity (95% CI)	0.80 (0.52–1.22)	1.31 (0.95–1.79)	1.74 (0.56–4.69)
Total	2873	3125	230
2012 (*n* = 6979)			
HIV positive	16	25	3
% positivity (95% CI)	0.51 (0.3–0.85)	0.70 (0.46–1.05)	1.06 (0.27–3.33)
Total	3146	3549	282

Total HIV positives	110	150	19
% positivity (95% CI)	0.76 (0.63–0.92)	0.94 (0.8–1.11)	1.64 (1.02–2.6)
Total	**14446**	**15993**	**1161**

^*^CI: confidence interval.
